# Estimation of Transmission of Porphyromonas Gingivalis from Mother to Child through Saliva

**DOI:** 10.31729/jnma.3475

**Published:** 2018-08-31

**Authors:** Khushbu Adhikari, Charanjeet Singh Saimbi, Birendra Prasad Gupta

**Affiliations:** 1 Department of Periodontology and Oral Implantology, College of Dental Surgery, Universal College of Medical Sciences, Bhairahawa, Nepal; 2 Central Department of Biotechnology, Tribhuvan University, Kathmandu, Nepal

**Keywords:** *chronic periodontitis*, *pregnancy*, *Porphyromonas gingivalis*, *polymerase chain reaction*, *saliva transmission*

## Abstract

**Introduction:**

Chronic periodontitis is an infectious disease. Porphyromonas gingivalis is the major pathogen associated with it and can be found in all ecosystems in the oral cavity. The presence of this organism is highly correlated with preterm and low birth weight babies. So, this study aimed to assess vertical transmission of P.gingivalis from pregnant women to their new born.

**Methods:**

Forty six pregnant women with chronic periodontitis were recruited for this cross-sectional study. Whole unstimulated saliva was collected from them before delivery and from their new-borns within forty eight hours of birth. Quantification of P.gingivalis in the saliva samples was carried out by quantitative real time polymerase chain reaction. The obtained data were analysed by SPSS 16 program.

**Results:**

The results showed a significant correlation (P=0.002) between the number of P.gingivalis present in the mother's saliva with that of the new-borns' saliva. DNA copies of more than 5000/ |il of P.gingivalis was found in 20 (43.5%) maternal saliva and 21 (45.7%) in new-borns' saliva. Both Plaque index and Extent and Severity index showed no correlation (P>0.05) with DNA copies of P.gingivalis in new-borns' saliva.

**Conclusions:**

The DNA copies of P.gingivalis found in new-borns' saliva are in par with mother saliva, as the saliva sample obtained from new-borns' were within forty eight hours of birth, no other environmental factor can have a direct role in its transmission. Thus, it can be concluded that P.gingivalis is vertically transmitted from mother to child.

## INTRODUCTION

Chronic periodontitis is an irreversible disease of the supporting structures of the tooth affecting the human population of all age.^1^ It is mainly associated with a gram negative obligate anaerobic organism Porphyromonas gingivalis (P.ginigvalis).^2^ This organism can be found in saliva, on the dorsum of the tongue, tonsils, buccal mucosa and gingiva, and other mucous membranes of patients suffering from chronic periodontitis.^3^

This organism has been found to play a role in the development of systemic diseases such as coronary heart disease, stroke and diabetes mellitus as well as preterm delivery of low birth weight infants.^4,5^

The first presence of bacteria in the mouth of a child can be found as soon as the beginning of the birth and is derived from the mother. It is a well-established fact that periodontal pathogens cluster in families and horizontal transmission of P. gingivalis between spouses, between caregivers and children has been suggested.^6,7^

Hence, this study aimed to assess vertical transmission of P. gingivalis from mother suffering from chronic periodontitis to her child.

## METHODS

This cross-sectional observational study was conducted by the Department of Periodontology and Oral Implantology, College of Dental Surgery, Universal College of Medical Sciences, Bhairahawa, including pregnant women admitted for normal delivery in the Department of Obstetrics and Gynaecology, Universal College of Medical Sciences from April 2013 to October 2014.

Ethical approval was obtained from the institutional review committee, Universal College of Medical Sciences, Bhairahawa. The informed written consent was taken from the participants. Pregnant women with chronic periodontitis at 37 to 40 weeks of gestation, were included in this study. Pregnant women with a history of smoking or with any systemic diseases and on any kind of medications or who had undergone oral prophylaxis six months prior to admission were excluded from the study.

A total of 46 pregnant subjects along with their new borns were enrolled in the study. To determine the sample size, a pilot study was carried out to identify the population of pregnant women in Lumbini zone of Nepal in the last five years from the records of different maternity hospitals. The sample size was calculated using an online calculator at 95% level of confidence and 14% margin of error.

A complete periodontal examination was carried out in the pregnant women by a single examiner. Plaque Index (Silness and Loe) was recorded and categorised as mild (0–0.9), moderate (1–1.9) and severe (2–3) for all subjects.^8^ Extent and Severity index by Carlos, which gives binominal scores of extent in percentage and severity in maximum probing depth was also recorded.^9^

Saliva contains a wide spectrum of molecules and microbes and has been used as a diagnostic fluid in medicine.^10^ Whole saliva samples have been reported as an excellent alternative to sampling individual periodontal pockets to detect P. gingivalis along with other periopathogens in the oral cavity.^11^ Several of the known periodontopathogens have similar percentage of DNA count in saliva compared to supra and subgingival tooth surfaces.^12^ Based on the above scientific background and ease of collection, saliva was selected as the most reliable medium for assessment of P.gingivalis in our study.

One ml of unstimulated whole saliva was collected in the morning hours using a calibrated micropipette from pregnant women in an upright position and was transferred into cryogenic vial of 1.8 ml capacity. The unstimulated saliva from their new-borns was collected taking extreme precaution against contamination within forty eight hours of birth and stored in vials as well. The samples were transported in an ice box with gel packs and stored at -20°C at the District Public Health Office, Bhairahawa, Nepal. The stored samples were transported to the National Public Health Laboratory, Teku, Kathmandu, Nepal for quantitative real time-polymerase chain reaction analysis.

The samples were first thawed to room temperature for the DNA extraction procedure. The procedure of DNA extraction was carried out using the QIAGEN DNA minikit (QIAGEN, Germany) following the manufacturer's instructions. The standard kit for detection and quantification of P. gingivalis from Primer Design Ltd. (Southampton, UK) was used. Real-time polymerase chain reaction was carried out using Rotor- Gene 6000 Series Thermocycler (Corbett Life Science, Sydney, Australia). The obtained number of amplified DNA copies was recorded in the subject's proforma and data was entered in Microsoft Excel.

The statistical analyses were carried out using Statistical Package for Social Sciences (SPSS) software program version 16.0. All continuous variables were presented as the mean±SD. Demographic variables were presented as frequency (%). The relationship between DNA copies of P.gingivalis in mother's saliva and child's saliva were examined by Pearson correlation analysis and multiple regression analysis was used to find independent determinants of DNA copies of P.gingivalis in child's saliva. A P value less than 0.05 were considered statistically significant.

## RESULTS

The results are summarised in tables 1 to 5. The demographic characteristics of the pregnant women included in this study are shown in Table 1. The mean age and haemoglobin level of pregnant women are 24.15±4.69 years and 11.05±1.74 mg/dl respectively.

**Table 1:** Description of the general characteristics of pregnant women.

Characteristics	n (%)
**Age group**	
17–20	11 (23.9)
21–30	31 (67.4)
31–40	4 (8.7)
**Socioeconomic status**	
Lower class	2 (4.3)
Lower middle class	5 (10.9)
Middle class	34 (73.9)
Upper middle class	4 (8.7)
Upper class	1 (2.2)
**Education**	
Uneducated	14 (30.4)
Middle school	14 (30.4)
High school	17 (37.0)
Master degree	1 (2.2)
**Number of meals**	
One	0 (0.0)
Two	5 (10.9)
Three	38 (82.6)
Four	3 (6.5)
**Type of diet**	
Mixed	44 (95.7)
Vegetarian	2 (4.3)
**Brushing aid**	
Tooth powder	1 (2.2)
Tooth brush	45 (97.8)
Hemoglobin (gm/dl)^*^	11.05±1.74

* *Values are presented in Mean±SD*

Plaque index was categorized as mild, moderate and severe. Of the total pregnant women, 27 (58.69%) had moderate plaque score with an overall mean plaque score of 1.39±0.57 (Table 2). The Severity and Extent index scores showed 31 (67.39%) subjects had mild periodontitis while the 34 (73.9%) were suffering from localized periodontitis (Table 2).

**Table 2:** Periodontal status of pregnant women.

Parameters	n (%)
**Plaque Index**	
0.0–0.9 (Mild)	10 (21.74)
1.0–1.9 (Moderate)	27 (58.69)
2.0–3.0 (Severe)	9 (19.56)
**Extent Index (%)**	
<30	34 (73.9)
>30	12 (26.1)
**Severity Index (mm)**	
1–2	31 (67.39)
3–4	13 (28.26)
>4	2 (4.34)

The salivary levels of DNA copies of P.gingivalis were assessed using quantitative real-time PCR. P.gingivalis was detected in all the samples. Twenty saliva samples (43.5%) from mother and twenty one saliva samples (45.7%) from new-borns showed DNA copies of P.gingivalis more than 5000//vl with a 2.2% increase when compared to the DNA copies in the mother's saliva to that of her new-born (Table 3). This inferred that saliva of the new born showed DNA copies of P.gingivalis similar to that of their mother.

**Table 3:** Real time PCR result for DNA copies of P.gingivalis in maternal and child's saliva.

DNA copies/*µ*l of P.gingivalis	DNA copies/*µ*l of P.gingivalis in Maternal saliva n (%)	DNA copies/*µ*l of P.gingivalis in Child's saliva n (%)	% change
<1000	3 (6.5%)	2 (4.3%)	−2.2%
1001–2000	0 (0%)	2 (4.3%)	4.3%
2001–3000	0 (0%)	1 (2.2%)	2.2%
3001–4000	1 (2.2%)	1 (2.2%)	0.0%
4001–5000	2 (4.3%)	0 (0%)	−4.3%
>5000	20 (43.5%)	21 (45.7%)	2.2%
NA	20 (43.5%)	19 (41.3%)	−2.2%
Total	46 (100%)	46 (100%)	–

We found a positive correlation (r = 0.44, P = 0.002) between DNA copies of P.gingivalis in maternal saliva and child's saliva (Table 4).

**Table 4:** Correlation of DNA copies of P.ginigvalis in child's saliva with clinical parameters and DNA copies of P.gingivalis in Maternal saliva.

Parameters	r value	P value
Plaque Index	0.155	0.304
Extent Index	0.025	0.868
Severity Index	−0.164	0.276
DNA copies of P.gingivalis in maternal saliva	0.448^*^	0.002

* *Correlation is significant at the 0.01 level (2-tailed).*

Further, multiple regression analysis also showed DNA P.gingivalis in maternal saliva is an independent predictor (ß = 0.45, P = 0.003) of P.gingivalis in child's saliva (Table 5).

**Table 5:** Independent predictors of DNA copies of P.ginigvalis in child's saliva.

Parameters	Beta	P value
Plaque Index	−0.224	0.301
Extent Index	0.371	0.099
Severity Index	−0.094	0.617
DNA copies of P. gingivalis in maternal saliva	0.458	0.003

## DISCUSSION

The early childhood years are critical period for acquisition of certain bacteria from parents or someone who had close household contact. Most common cause attributed to the transmission of pathogens has been the lower genital tract during labour and also breastfeeding. But for the transmission of oral pathogens, salivary contact through daily activities such as talking, kissing, tasting the food seems more probable and has been studied upon. Parents with severe chronic periodontitis may serve as a source of infection to their children and put them at a higher risk of acquiring pathogens responsible for periodontitis.^13^ The results of our study shows the number of P.gingivalis in saliva of the new-borns were similar to that found in saliva of their respective mother. Steenbergen et al, also indicated the possibility of transmission of P. gingivalis between spouses on the basis of its presence in saliva samples.^7^

The socioeconomic status helps us to know their accessibility to various medical and dental care for themselves and their children. In this study, 89.1% of women belonged to middle and lower class. The mean haemoglobin levels of most of the pregnant women were less than 12 mg/dl (Table 1). This may be due to an increased requirement of iron during pregnancy.^14^ The need of iron, folic acid and vitamin B12 supplements during pregnancy are highlighted.

On assessing the clinical parameters, moderate plaque index score was observed in most of the pregnant women, (Table 2) as scrub technique of tooth brushing was a common practice among them and use of any other oral hygiene aids such as dental floss was not noted. Plaque starts to build up immediately after tooth-brushing,^15^ hence, to maintain a good oral hygiene, brushing on a daily basis helps to keep plaque under control. Increased Plaque index score in pregnancy can be attributed to stress, fluctuating hormone levels and physical discomfort.^16,17^ Localized mild periodontitis was prevalent in our study. Pregnancy itself does not cause periodontal disease but provides a suitable environment for bacterial growth which predisposes women to periodontal diseases. Many women go through their pregnancies without giving much consideration to their oral health status which should be a focus along with other aspects of health at every antenatal visit.^18^ This study highlights the importance of regular dental service during pregnancy and asserts that women are more likely to use dental services during pregnancy if they were aware about the possible connection between oral health and pregnancy outcome.^19^

The finding in this study revealed that the concentration of P.gingivalis in saliva sample of new born child was found to be correlating with the concentration in the saliva sample of the mother. From this, we may conclude that clinically similar strains of microorganisms can be transferred from mother to her new born child in a recognizable amount. However, results from family studies suggest that environmental factors appear to be the major determinants of variance in chronic periodontitis whereas data from twin studies indicate that both genetic and environmental factors influence the bacterial colonization and disease.^20,21^

It was observed that the number of P. gingivalis in maternal saliva had a direct influence on the number of P. gingivalis in saliva of the new born child rather than the extent and severity of periodontal disease in the mother. Hence, we could say that periodontal status of the mother is not a determinant of the periodonto pathogen in the children, which is supported by the results of the study by Yang et al.^22^ The results of the present study have led us to suppose that the maternal saliva may act as a source of gram negative anaerobes like P. gingivalis in the oral micro flora of edentulous infants.^23^ The role of the father and other family members is also equally important and should also be considered. However, the transmission does not guarantee colonization until a suitable habitat for colonization of these organisms is present, which may not occur until after tooth eruption.

A cross-sectional study design limited examination of the same mother-infant pairs at successive time periods. Also, the presence and quantification of P. gingivalis in the amniotic fluid samples of the mother could have added more meaning to the results. Further longitudinal studies to confirm the role of salivary transmission with the use of more specific molecular methods is desirable as the next step in understanding the exact pattern of transmission. The results of which will highlight the preventive aspect in the field of chronic periodontitis, starting from recognizing the target population who are more prone to this destructive disease. As it is well known fact for any kind of disease that “prevention is better than cure.”

## CONCLUSIONS

DNA copies of P. gingivalis found in saliva of child are identical with saliva of mother. As the saliva sample of the new born child were collected within 48 hours of birth and no other environmental factor can have a direct role in transmission, it is clear that the DNA copies of P. gingivalis were vertically transmitted from mother to child.
